# Determination of Endogenous Bufalin in Serum of Patients With Hepatocellular Carcinoma Based on HPLC-MS/MS

**DOI:** 10.3389/fonc.2019.01572

**Published:** 2020-01-23

**Authors:** Mengfei Han, Geliang Yang, Qing Lin, Yanlong Yang, Huiqing Zhang, Yonghua Su

**Affiliations:** ^1^Department of Traditional Chinese Medicine, Changhai Hospital, Naval Medical University, Shanghai, China; ^2^Department of Traditional Chinese Medicine and Acupuncture, The Second Medical Centre, Chinese People Liberation Army General Hospital, National Clinical Research Center for Geriatric Diseases, Beijing, China; ^3^Fuzhou Traditional Chinese Hospital, Fuzhou, China; ^4^Department of Dermatology, Yueyang Hospital of Integrated Traditional Chinese and Western Medicine, Shanghai University of Traditional Chinese Medicine, Shanghai, China

**Keywords:** endogenous bufalin, HCC, HPLC-MS/MS, cardiotonic steroid, serum

## Abstract

Bufalin is a cardiotonic steroid and a key active ingredient of the Chinese medicine *ChanSu*. It has significant anti-tumor activity against many malignancies, including hepatocellular carcinoma (HCC). Previous studies have shown that human bodies contain an endogenous bufalin-like substance. This study aimed to confirm whether the endogenous bufalin-like substances is bufalin and further detect the differences between HCC and control groups of endogenous bufalin concentration by the high-performance liquid chromatography coupled tandem mass spectrometry (HPLC-MS/MS). The results confirmed the endogenous bufalin-like substance is bufalin. Totally, 227 serum samples were collected: 54 from HCC patients and 173 from healthy volunteers constituting a control group. Both the test group and the control group contained bufalin in serum, revealing that bufalin is indeed an endogenous substance. The bufalin concentration was 1.3 nM in HCC patients and 5.7 nM in normal people (*P* < 0.0001). These results indicate that human bodies contain endogenous bufalin, and it may be negatively correlated with the incidence of HCC.

## Introduction

Hepatocellular carcinoma (HCC) is the most common type of liver cancer, which is the fourth most common malignancy and the third leading cause of cancer related deaths in China (1). Surgical resection is regarded as the only radical treatment of HCC. However, the prognosis of patients with HCC remains unsatisfactory due to carcinoma recurrence and a limited response to targeted therapy, chemotherapy, and radiotherapy (2). The small-molecule multikinase inhibitor sorafenib has been the only systemic therapy proven to extend overall survival when used as a first-line treatment over 10 years, showing a median improvement of 2.8 months compared with placebo, despite a low response rate of 2% (3). Recently, other small-molecule multikinase inhibitors (e.g., regorafenib and lenvatinib) have been approved for HCC treatment, but median survival time in patients was <13.6 months (4). Therefore, it is of great significance to explore new effective treatments of HCC.

Traditional Chinese medicines (TCM), including plants, animal parts, and minerals, have drawn a great deal of attention in recent years for their potential in the treatment of HCC and can prevent recurrence after resection of small HCC (5–7). In TCM practice, *Chansu* (venom of toad skin) and *Chanpi* (the skin of toad) have been used in the treatment of tumors, including HCC (8–10). Bufalin has been recognized as a prominent digoxin-like component and a potential Na^+^-K^+^-ATPase inhibitor from *Chansu* and *Chanpi* (11, 12). Recent studies proved that bufalin has marked anti-tumor activities through its ability to inhibit proliferation, induce apoptosis and autophagy, reverse drug resistance, and inhibit invasion and metastasis of HCC (13–21). Some scholars have used high-performance liquid chromatography to detect the content of bufalin in *Huachansu* preparations including injections, tablets, and capsules. The bufalin in these drugs that enters the human body by intravenous injection or oral administration is called exogenous bufalin.

Previous studies have demonstrated that there may be a new type of steroid hormone in healthy people. Such hormones include bufalin-like substance that were thought to exist only in amphibian toads. Ferrandi et al. ruled out the possibility that these substances were derived from food, confirming that this was an endogenous substance (22–24). Weidemann et al. detected the content of a digoxin-like substance in serum of 84 women with breast cancer and found that 73.6% of the patients had significantly lower levels of this substance than healthy people (25, 26). In 1995, Numazawa et al. extracted an endogenous bufalin-like substance from normal human plasma by separation, purification, and immunological methods. The endogenous bufalin-like substance was similar to the function of exogenous bufalin and could inhibit the growth of a variety of human leukemia cells, which suggested that this endogenous bufalin-like substance could act as an important player in inducing cell differentiation *in vivo* (27, 28). In 2001, Oda et al. (27) determined by means of monoclonal antibodies that the concentration of the bufalin-like component was mostly maintained at 5 nM in serum from 19 healthy volunteers.

This study aimed to confirm whether endogenous bufalin-like substances are bufalin, detect endogenous bufalin substances in the serum of HCC patients and healthy volunteers, and investigate the potential relationship between endogenous bufalin and the incidence of HCC.

High-performance liquid chromatography with tandem mass spectrometry (HPLC-MS/MS) is considered a powerful analytical tool increasingly applied to endogenous detection of hormones. In the early stage of the research group, HPLC-MS/MS was established to determine the concentration of bufalin in rats after intravenous administration. The methodological results of the determination of each biological sample show that the linearity, precision, and accuracy of the method are satisfactory (29). In this study, for the first time HPLC-MS/MS was used to qualitatively and quantitatively analyze and examine the differences of endogenous bufalin from HCC patients and healthy volunteers in serum.

## Materials and Methods

### Chemicals and Reagents

Bufalin (>98% purity) was purchased from Sigma-Aldrich Company (St. Louis, MO, USA). Cinobufagin [>97% purity, internal standard (IS)] was purchased from the National Institute for the Control of Pharmaceuticals and Biological Products of China (Beijing, China). All were corrected for purity and salt forms when weighed or diluted for standard stocks, whose chemical structures are shown in Supplementary Figure 1. HPLC-grade methanol and acetonitrile were obtained from Fisher Scientific Company (Pittsburgh, USA). Formic acid was purchased from MREDA Company (Beijing, China). Ultrapure water was produced by A. S. Watson (Guangzhou, China). All other reagents were of analytical grade.

### HPLC-MS/MS Instrument and Analytical Conditions

The HPLC-MS/MS system consisted of an Agilent 1200 series high performance liquid chromatograph (HPLC) and an Agilent 6410 triple quadruple mass spectrometer equipped with an electrospray ionization (ESI) source (Agilent Technologies, Santa Clara, CA, USA). Data were analyzed by Mass Hunter software (Agilent Corporation, Santa Clara, CA, USA). A Waters XSELECT™ HSS T_3_ column (100 mm × 2.1 mm, i.d., 2.5 μm) was used for chromatographic separation. The mobile phase was consisted of acetonitrile and 0.1% formic acid in water (65:35, v/v). The column was equilibrated and eluted at a constant flow rate of 0.3 mL · min^−1^, maintained at 35°C. The sample injection volume was 10 μL, and the run time was 3.0 min. Data acquisition was performed using multiple reaction monitoring (MRM) of bufalin with the corresponding IS. Transitions were monitored at m/z 387.3 → 255.3 for bufalin, and at m/z 443.2 → 365.1 for the IS (Supplementary Table 1, Supplementary Figure 2). The detection parameters were optimized as follows: drying gas temperature, 325°C; drying gas flow rate, 10.0 L · min^−1^; nebulizer pressure, 40 psi; capillary voltage, 4,000 V.

### Preparation of Bufalin Stock Solution and Quality Control Samples

The stock solution of bufalin was prepared separately in methanol-water (5: 95, V/V) solution at a concentration of 1.0 μg · mL^−1^. All working solutions were freshly prepared by serially diluting stock solutions with mobile phase.

### Sample Collection and Preparation

All serum samples were separated from the clotted whole blood by centrifugation at 3,000 rpm for 15 min. A 1 mL aliquot of each serum sample was collected and stored at −80°C until analysis. Before analysis, all serum samples were simultaneously thawed at room temperature. A 100 μL aliquot was mixed with methanol (300 μL each) by vortexing, and the mixture was left on ice for 5 min. Samples were then centrifuged at 14,000 rpm for 10 min at 4°C. The supernatant was transferred to an autosampler vial, and 10 μL of the solution was injected into the analytical column separately for HPLC-MS/MS identification.

### Participants

This clinical trial was reviewed and approved by the Changhai Hospital Ethics Committee (CHEC2015-113). Approvals for the study protocol (and any modifications thereof) were obtained from independent ethics committees. Healthy volunteers and HCC patients were recruited for the study carried out in Changhai Hospital between May 2015 and December 2015. All subjects provided signed informed consent.

Men or women older than 18 years old were eligible to participate in the study as healthy volunteers. Healthy volunteers were excluded if they got pregnant or had infectious diseases including hepatitis, malignant tumors, or other major diseases such as liver and kidney failure.

Eligible patients were men or women older than 18 years old with hepatocellular carcinoma diagnosed according to the diagnostic criteria detailed in the Expert Consensus of the Standard Diagnosis of Primary HCC issued in 2011 (30). Patients who had previously used toad-related preparations (including interventional, intravenous, oral, and topical routes), taken anti-tumor Chinese medicines or hormonal drugs, were critically ill, had other tumors, did not return after HCC surgery, had legal infectious diseases (except viral hepatitis), or had mental disorders were excluded from the study.

### Method Validation

Method validation including determination of specificity, precision, and accuracy, extraction recovery, matrix effect, and stability was performed according to the Chinese pharmacopeia (Version 2010).

For specificity, comparison of responses in spiked and blank samples from at least 6 lots was performed. A false positive rate of <20% was considered acceptable. Extraction recovery and matrix effect were assessed in three replicates at three concentration levels (low, mid, and high) for bufalin (2.0, 10.0, and 50.0 ng/mL). The matrix effect was the ratio of peak area in the spiked post-extraction samples to the concentration of the corresponding solvent substituted samples, and the recovery was the ratio of peak area in the spiked samples concentration to that of the corresponding spiked post-extraction samples.

Inter-day and intra-day precision and accuracy were assessed in five replicates at three concentration levels (low, mid, and high). Samples were analyzed in three analytical lots in separate days (at least 2 days), and the RSD% (relative standard deviation) for inter-day and intra-day precision of no more than 15% was satisfactory. For intra-day and inter-day accuracy, RE% (relative error) within 15% was considered acceptable.

Stability, including long term stability (12 h at room temperature), short term stability (6 h in an autosampler), and three freeze-thaw cycle stability, was evaluated using quality control (QC) samples at the same three concentration levels.

### Statistical Analysis

All data were calculated from the concentration of bufalin using Mass Hunter software. Data are expressed as mean ± standard deviation (SD) and SPSS Version 18.0 statistical software (SPSS, lnc., Chicago, IL, USA) was used to process all the data. For comparisons, chi-squared test, Dunnett's test, Wilcoxon signed-rank test, and Mann–Whitney *U*-test were performed, as appropriate. *P* < 0.05 was taken as statistical significance.

## Results

### Specificity

**Figure 1:**
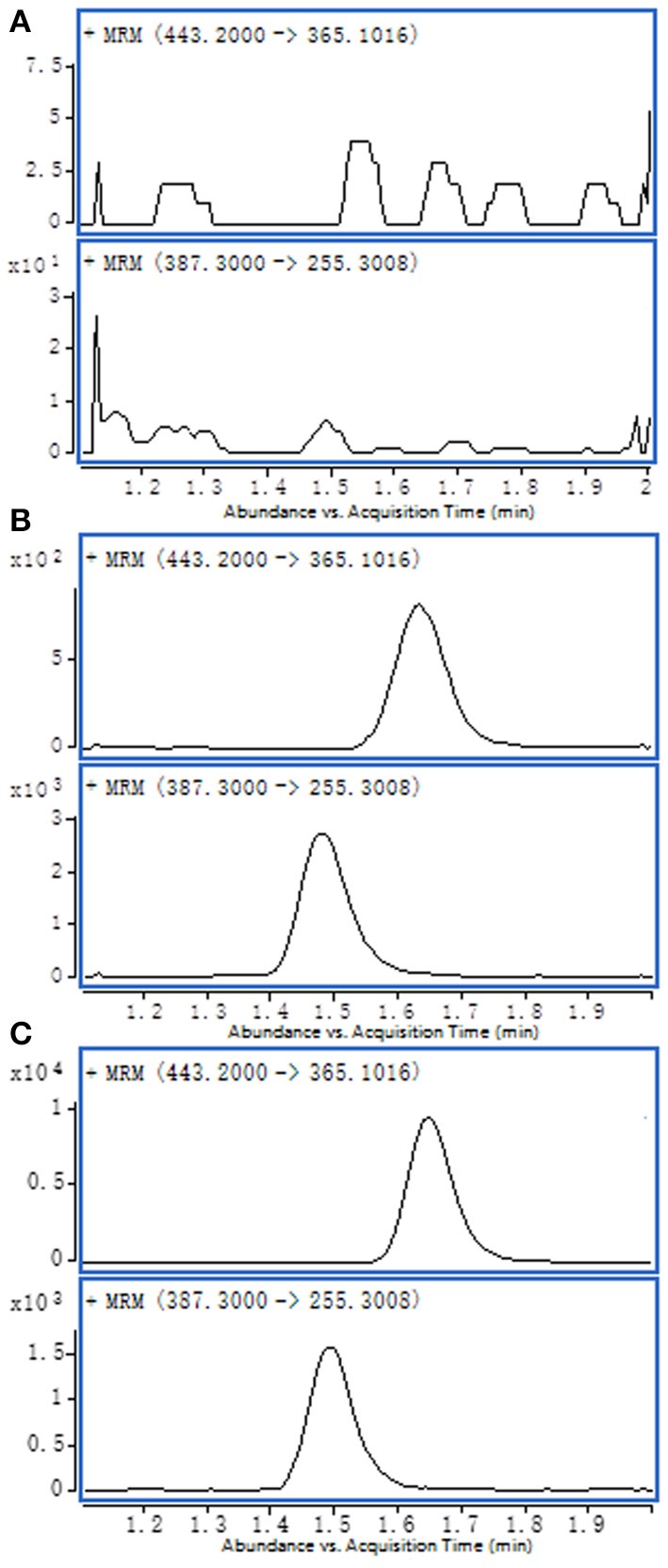
Representative MRM chromatograms: **(A)** blank serum sample; **(B)** water spiked with bufalin and IS; **(C)** test serum sample.

Comparing the blank matrix and water spiked with bufalin and IS from the chromatograms, no significant interferences can be seen at any given retention time; this indicated that human serum contains bufalin. A good baseline separation was achieved for each component (shown in Figure 1).

### Assay Precision and Accuracy

Intra-day and inter-day precision and accuracy for bufalin from human serum samples data are shown in Supplementary Table 2. All intra-day and inter-day precision and accuracy were acceptable with RSD% <15% and RE% within ±15%.

### Extraction Recovery and Matrix Effect

The extraction recoveries of the bufalin under the protein precipitation conditions are summarized in Supplementary Table 3. The extraction recoveries and matrix effect of bufalin at the three concentration levels in serum were 91.4–96.8% and 84.6–98.9%, respectively.

### Stability

The stability results (Supplementary Table 4) showed that bufalin, at the three concentrations studied, had acceptable stabilities after three freeze-thaw cycles, at room temperature (20°C) for 12 h, and in and auto-sampler (4°C) for 6 h after protein precipitation.

### Subjects' Characteristics

After screening, 173 healthy volunteers and 54 HCC patients were enrolled. The ages of healthy volunteers ranged from 20 to 92 years (mean ± SD, 46.9 ± 11.8) and HCC patients ranged from 26 to 77 years (mean ± SD, 55.4 ± 11.2). The characteristics of HCC patients in terms of age, sex, alpha-fetal protein (AFP) levels, total bilirubin (TB), alanine transaminase (ALT) levels, and the Barcelona clinic liver cancer (BCLC) stage are summarized in Table 1.

**Table 1:** Subjects' characteristics.

**Characteristic**	**Healthy volunteers (*n* = 173)**	**HCC patients (*n* = 54)**	**Total (*n* = 227)**
**SEX**			
Male	123 (71.1%)	49 (90.7%)	172 (75.8%)
Female	50 (28.9%)	5 (9.3%)	55 (24.2%)
**AGE GROUP (YEARS)**			
<40	60 (34.7%)	3 (5.6%)	63 (27.8%)
≥40–<60	82 (47.4%)	32 (59.3%)	114 (50.2%)
≥60	31 (17.9%)	19 (35.2%)	50 (22.0%)
**BARCELONA CLINIC LIVER CANCER STAGE**			
A		11 (20.4%)	
B		9 (16.7%)	
C		33 (61.1%)	
D		1 (1.9%)	
**ALPHA-FETAL PROTEIN**			
<10		23 (57.4%)	
≥10		31 (42.6%)	
**HEPATITIS B SURFACE ANTIGEN**			
+	0 (0)	54 (100%)	
−	173 (100%)	0 (0)	
**TOTAL BILIRUBIN**	11.4 ± 5.5	22.9 ± 15.3	
**ALANINE TRANSAMINASE**	21.1 ± 10.4	68.4 ± 45.4	

According to the American Association for the Study of Liver Diseases (AASLD) practice guidelines for the management of HCC, the patients with BCLC stage A (11 cases) received resection and had recurrence after surgery. Patients with BCLC stages B-D (43 cases) received transcatheter arterial chemoembolization (TACE) treatment. No patients received targeted therapy, chemotherapy, or radiotherapy.

### Healthy Volunteers' Serum Contains Endogenous Bufalin

The median concentration of bufalin in the serum of healthy volunteers was 2.2 ng · mL^−1^ (5.7 nM), which was consistent with the study by Miwa Oda (5 nM) (27). There was a statistically significant difference between male and female (Supplementary Table 5; *P* = 0.016). And there were also significant differences in serum bufalin concentration between the <40, ≥40–<60, ≥60 years age groups in healthy volunteers (Supplementary Table 5; *P* = 0.007).

### Serum of Patient With HCC Contained Endogenous Bufalin

The median bufalin concentration in patients with HCC was 0.5 ng · mL^−1^ (1.3 nM). There was a significant difference in serum bufalin concentrations between healthy volunteers and liver cancer patients (Table 2; *P* < 0.0001). Compared with the healthy group, there was no significant difference in bufalin levels between males and females (Supplementary Table 5; *P* = 0.45) or between the <40, ≥40– <60, and ≥60 years age groups in patients with HCC (Supplementary Table 5; *P* = 0.11).

**Table 2:** The comparison of endogenous bufalin concentration between healthy volunteers and hepatocellular carcinoma patients.

**Group**	**Bufalin concentration (ng · mL**^****−1****^**)**		***P***
	**(mean ± SD)**	**median (min, max)**	
Health	2.7 ± 1.8	2.2 (0.2, 10.7)	<0.0001
Hepatocellular carcinoma	0.7 ± 0.8	0.5 (0.1, 3.6)	

### The Relationship Between Endogenous Bufalin and AFP Levels in HCC Patients

We also investigated the relationship between endogenous bufalin and AFP levels in HCC patients. In the low AFP (<10 ng/mL) group, the median bufalin concentration was 0.3 ng · mL^−1^ (0.8 nM). In the elevated AFP (≥10 ng/mL) group, the median bufalin concentration was 0.5 ng · mL^−1^ (1.3 nM). There was no significant difference in bufalin levels between low AFP and elevated AFP groups in HCC patient (Table 3; *P* = 0.56).

**Table 3:** The relationship between endogenous bufalin and alpha-fetal protein levels in HCC patients.

**Alpha-fetal protein levels**	**Bufalin concentration (ng · mL**^****−1****^**)**		***P***
	**(mean ± SD)**	**median (min, max)**	
<10	0.7 ± 0.8	0.3 (0.1, 3.3)	0.56
≥10	0.8 ± 0.7	0.5 (0.1, 3.6)	

## Discussion

The endogenous digitalis-like compounds (EDLC), a group of steroids, have been demonstrated to exist in mammals as potential Na^+^/K^+^-ATPase inhibitors (31–35). These compounds are postulated to play an essential role in the pathophysiology of hypertension, preeclampsia, end-stage renal disease, congestive heart failure, and diabetes mellitus (36). Bufalin is a cardiac glycoside steroid, which have anti-carcinoma, anti-inflammatory, and immune-regulating effects, similar to steroid hormones (37). Consistent with previous studies (27), healthy volunteers' serum contains endogenous bufalin-like substance. The major finding in this study is confirmation that the endogenous bufalin-like substance is bufalin. The concentration (5.7 nM) is consistent with the previous data (5 nM) in healthy volunteers. We further found that the endogenous bufalin concentration in HCC patients is significantly reduced.

In healthy volunteer group, the concentration of bufalin in males was significantly higher than in females. Although the endocrine system in both men and women is regulated by the hypothalamic-pituitary-adrenal (HPA) axis, there are still differences in hormone types and secretion levels between males and females. In addition to the currently known glucocorticoids, sex hormones, and mineralocorticoids, there are likely other structurally similar steroidal hormones in the body (22); bufalin may be one of them. Our results suggest that secretion of bufalin may be similar to the pattern of secretion of other hormones in the human body. In the <40 age group, the secretion of bufalin gradually increases, reaching a peak in the 40–59 years old group. After 60 years old, production of bufalin drops rapidly, and its concentration is even lower than in 20–39 years old cohort.

Low EDLC plasma concentrations may significantly increase an individual's risk of developing cancer (26). Weidemann et al. (38) compared EDLC plasma and cortisol serum concentrations in breast cancer patients (*n* = 22) and patients with a benign breast disease (*n* = 10) and found than there was a significant positive correlation between EDLC and cortisol in the control as well as in patients (rs = 0.7, *P* = 0.05). They hypothesized that a lowered EDLC response threshold of tumor as compared with normal cells increases the risk of tumorigenesis, especially in individuals with reduced EDLC plasma concentrations after long stress exposure (38). Previous studies have demonstrated that plasma concentration of EDLC, including bufalin-like substances, in patients with breast cancer or leukemia were reduced compared to healthy people (28). In our study, endogenous bufalin did show significant down-regulation in HCC patients as compared with healthy volunteers (*P* < 0.0001). Therefore, we speculate that the decrease in bufalin concentration may be related to the occurrence and development of certain tumors, but whether the endogenous bufalin itself has anti-tumor effects was not covered in this experiment. By detecting endogenous bufalin in healthy volunteers and HCC patients, we are able to propose the following points: (1) The human body produces hormones which promote cell differentiation, induce apoptosis and prevent the occurrence of tumors, and bufalin may be one of them; (2) In the event of viral or aflatoxin infection, emotional depression, fatigue, or another condition, the levels of such hormones may be altered; this alteration could have an important correlation with the occurrence and development of HCC.

AFP, as a tumor marker, is widely used clinically for the diagnosis and screening of HCC for many years (39). However, it has been recognized that AFP is less sensitive in detecting HCC and that AFP levels usually increase in other cases of liver disease (chronic hepatitis or cirrhosis) without HCC (40, 41). Our study demonstrated that there was no direct relationship between endogenous bufalin concentration and AFP levels in HCC patients. The endogenous bufalin may be used as a supplement to AFP for clinical diagnosis of HCC in the future so that more patients can benefit from the optimal therapy.

Finally, there are also some limitations in the present study. Our results need to be corroborated by more evidence that bufalin concentration is negatively associated with the incidence of HCC. As this is a single-center, small-size study, we don't know whether the conclusion could be extended to different stages and histological types of HCC. Future studies of the association of endogenous bufalin with the development of HCC considering more histological types of HCC with multi-centered data collection should be carried out to confirm the hypothesis. Moreover, patients with HCC should be dynamically tested for changes in endogenous bufalin based on changes in their condition, and animal studies on supplementation of exogenous bufalin to inhibit the occurrence of HCC could be conducted.

## Conclusion

In this study, we determined by HPLC-MS/MS that healthy volunteers and HCC patients both produce endogenous bufalin, and for the first time confirmed that the bufalin concentration in patients is generally lower than that of healthy people. Bufalin may have a negative correlation with the incidence of HCC.

## Data Availability Statement

The datasets generated for this study are available on request to the corresponding author.

## Ethics Statement

The studies involving human participants were reviewed and approved by Changhai Hospital Ethics Committee. The patients/participants provided their written informed consent to participate in this study.

## Author Contributions

YS and HZ contributed conception and design of the study. MH, GY, and QL collected patient samples and tested serum samples. QL and YY performed the statistical analysis. All authors participated in the writing of the manuscript and confirmed the final review of the manuscript.

### Conflict of Interest

The authors declare that the research was conducted in the absence of any commercial or financial relationships that could be construed as a potential conflict of interest.
