# Supplementary material for: Determination of Endogenous Bufalin in Serum of Patients With Hepatocellular Carcinoma Based on HPLC-MS/MS
Source: Front Oncol. 2020 Jan 23;9:1572. doi: 10.3389/fonc.2019.01572 (PMC6989541; doi:10.3389/fonc.2019.01572)
Supplement: Supplementary file 1 [file Data_Sheet_1.pdf]

Supplementary Table 1. Optimized MS detection conditions

Analytes	Precursor ion(m/z)	Product ion(m/z)	Fragmentor energy(V)	Collision energy(eV)	Polarity
Bufalin	387.3	255.3	85.0	22.0	Positive
Cinobufagin (IS)	443.2	365.1	135.0	18.0	Positive

Supplementary Table 2 . Intra-day and inter-day precision and accuracy of bufalin in human serum(n = 5)

Concentration added (ng/mL)	Intra-day			Inter-day		
	Measured concentration (mean ± SD, ng/mL)	Precision RSD%	Accuracy RE%	Measured concentration (mean ± SD, ng/mL)	Precision RSD%	Accuracy RE%
2	2.2±0.1	2.8	10.6	2.0±0.2	9.2	8.1
10	10.4±0.7	7.0	3.8	10.3±0.7	7.0	6.2

50	53.9 ±2.4	4.4	7.7	51.1±3.8	7.5	6.6

RE is expressed as [(mean measured concentration)/(spiked concentration) − 1] × 100%

Supplementary Table 3. The recovery and matrix effect of bufalin in serum (n=3)

Concentration added (ng/mL)	Recovery(%)	CV(%)	Matrix effect(%)	CV(%)
2	94.9±1.8	1.9	84.6±4.4	5.2
10	91.4±7.5	8.2	98.9±4.6	4.6
50	96.8±1.8	1.9	96.2±5.0	5.2

Supplementary Table 4 . Stability of bufalin at different QC levels(n = 3)

Analyte	Concentration added (ng/mL)	After three freeze-thaw cycles		At room temperature（20°C） for 12h		At auto-sampler（4°C） for 6h	
		Measured concentration (mean ± SD, ng/mL)	Precision RSD%	Measured concentration (mean ± SD, ng/mL)	Precision RSD%	Measured concentration (mean ± SD, ng/mL)	Precision RSD%

Bufalin	2	2.0±0.1	5.0	1.9±0.1	3.6	2.2±0.2	2.0±0.1
	10	9.9±0.6	5.7	10.1±0.7	6.6	10.1±0.7	9.9±0.6
	50	55.2±1.1	1.9	49.2±5.7	11.6	53.1±4.9	55.2±1.1

Supplementary Table 5 . The endogenous bufalin concentration in healthy volunteers and HCC patients

Characteristic	Healthy volunteers		HCC patients	
	Bufalin concentration（ng · mL^{-1}） (mean ± SD) median（min, max）	*P-Value*	Bufalin concentration（ng · mL^{-1}） (mean ± SD) median（min, max）	*P-Value*
Sex				
Male	2.9±1.4 2.3(0.2,10.7)	0.016	0.8±0.8 0.5（0.1,3.6）	0.45
Female	2.2±1.3 1.9(0.2,7.0)		0.5±0.6 0.3（0.1,1.5）	
Age group（years）				
<40	2.8±1.7 2.3(0.6,7.9)	0.007	0.2±0.03 0.2(0.1,0.2)	0.11
≥40 to <60	2.9±1.9 2.6(0.2,10.7)		0.8±0.9 0.5(0.1,3.6)	

≥60	2.0±1.0	1.7(0.2,5.9)		0.5±0.5	0.6(0.1,2.0)
