# Supplementary figures and images for: Determination of Endogenous Bufalin in Serum of Patients With Hepatocellular Carcinoma Based on HPLC-MS/MS
Source: Front Oncol. 2020 Jan 23;9:1572. doi: 10.3389/fonc.2019.01572 (PMC6989541; doi:10.3389/fonc.2019.01572)

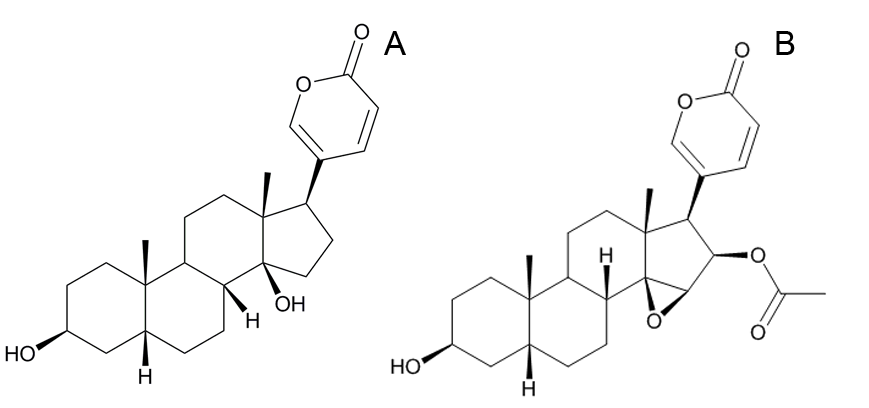

Supplement: Supplementary file 2 [file Image_1.TIF]

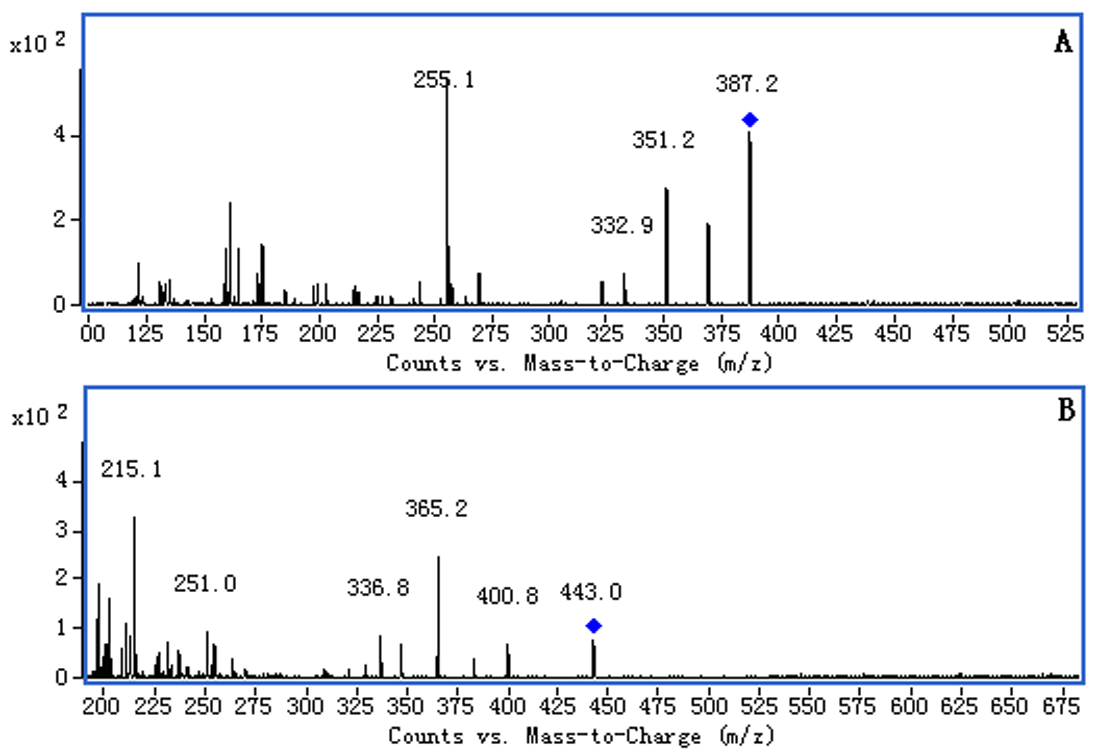

Supplement: Supplementary file 3 [file Image_2.TIF]
